# An assessment of the effect of the genotype on postoperative venous thromboembolism risk in 140,831 surgical patients

**DOI:** 10.1016/j.amsu.2021.102938

**Published:** 2021-10-13

**Authors:** Mathias A Christensen, Alexander Bonde, Martin Sillesen

**Affiliations:** a Department of Surgical Gastroenterology and Transplantation C-TX, Copenhagen University Hospital, Rigshosptialet, Denmark; b Center for Surgical Translational and Artificial Intelligence Research CSTAR, Copenhagen University Hospital, Rigshospitalet, Denmark; c Institute of Clinical Medicine, University of Copenhagen, Denmark

**Keywords:** Venous thromboembolism, SNP, Biobank, Personalized medicine, Surgery, *ABO*

## Abstract

**Background:**

Postoperative Venous Thromboembolism Events (VTE) constitute a major source of morbidity and mortality after surgery. The aim of this study was to investigate whether commonly occurring Single Nucleotide Polymorphisms (SNPs) are associated with VTE in the surgical setting.

**Methods:**

Retrospective study using data from the United Kingdom (UK) biobank, a genome biobank containing healthcare and genotyping data from more than 500.000 individuals. A cohort of 140,831patients with a registered surgical procedure was identified and used for a discovery genome wide association study (GWAS), with the remainder of the cohort (305,349 non-surgical patients) used as a replication cohort. Primary outcome was associations between SNPs and VTE within 30 days after a surgical procedure. Genome wide significance was set at p = 5 × 10^−8^.

**Results:**

In the surgical (discovery) cohort, no SNPs reached genome wide significance. The VTE association of the top candidate SNP in the *ABO* gene rs505922 (p = 3.33 × 10^−7^), was replicated in the general (replication) cohort (p = 2.42 × 10^−59^).

**Conclusions:**

and Relevance: This study did not identify associations between SNPs and postoperative VTE events reaching genome-wide significance, although the VTE relevance of top candidates were demonstrated.

## Highlights

•Venous thromboembolisms are common after surgery.
•Large biobanks help identify common genetic risk factors.
•Common variants in ABO may increase the risk of postoperative venous thromboembolisms.
•Genotyping surgical patients may benefit preoperative risk assessment.

## 1 Introduction

Venous thromboembolism events ((VTE), both deep venous thromboses (DVT) and pulmonary embolism (PE)) remain a common cause of morbidity and mortality postoperatively [1,2]. Although being a common cause of death, with an estimated 150,000 to 200,000 annual deaths related to VTE in the United States alone, the risk can be reduced with appropriate thrombophylactic agents, which should be considered in every surgical patient [3,4].

Several clinical features have been associated with a higher risk of VTE including increasing age, malignancy, oral contraception, estrogen replacement therapies, obesity, prior VTE, trauma as well as the surgical intervention in itself [5,6]. Furthermore, procedure related risk factors include long duration of surgery, emergency surgery, type of anesthesia, positioning of the patient and prolonged postoperative immobilization [[7], [8], [9]]. As Virchow's triad emphasizes, hypercoagulability contributes to thromboses [10], which may be aggravated by factors including specific genotype variations like the factor V Leiden mutation [11,12]. Genetic risk factors besides factor V Leiden are increasingly being recognized as significant contributors to the risk of VTE, with several Genome-Wide Association Studies (GWAS) having identified multiple single nucleotide polymorphism (SNP) loci associated with an increased risk of VTE in the general population. Klarin et al. identified loci with the Million Veteran Program and United Kingdom (UK) Biobank data, with the factor V Leiden variant rs6025 being the top candidate as well as discovering and replicating 22 candidate loci associated with VTE [13]. Some of the identified loci have previously been reported in a meta-analysis by Germain et al. with loci near genes *F5*, *F2*, *ABO* and *FGG* being the major replicated candidates for increased VTE risk, as well as three new loci near *ZFPM2, SLC44A2* and *TSPAN15* [14].

As surgery is a major risk factor for VTE events, it is essential to identify patients with an increased risk of postoperative VTE. Although multiple SNPs are associated with VTE in the general population, it is currently unknown whether the surgical stress response influences these associations and consequently how these SNPs contribute to the postoperative VTE risk, which constitutes the primary focus of this study. Secondly, it is unknown whether genomic VTE risk factors in the general population can be directly translated to the surgical setting. We hypothesized that commonly occurring single nucleotide polymorphisms could be associated with altered 30-day risk of a postoperative VTE event, and that an overlap between top genomic variations in both a surgical and non-surgical cohort could be identified.

## 2 Methods

### 2.1 Disease phenotype data

Access to the UK biobank data was approved by the UK biobank consortium (Study ID #60861). Data is reported in accordance with the Strengthening the reporting of cohort studies in surgery (STROCSS) guidelines [15]. The study was registered at clinicaltrials.gov (#NCT04940377). We conducted a two-stage GWAS for the risk of postoperative VTE. First, a discovery GWAS-analysis was performed to identify VTE-risk SNPs in a cohort of surgical patients. Postoperative VTE was defined as a VTE event occurring up to 30 days following a surgical procedure as recorded in the UK biobank dataset.

Secondly, in order to validate the association with VTEs as well as to explore whether there was an overlap between SNP associated VTEs in the surgical and non-surgical population, the analysis was replicated in a different cohort of the general, non-surgical population (replication GWAS). This approach was chosen, as different pathophysiological mechanisms for VTE may exist between surgical and non-surgical patients.

The analysis was conducted using a population-based cohort in the United Kingdom (UK) Biobank [16]. The UK Biobank is a national health resource of more than 500,000 individuals between the age of 40–69, recruited and included between 2006 and 2010, with prospective follow up for at least 30 years. Participants were originally identified and invited from National Health Service registries and asked to fill out an automated questionnaire about lifestyle and medical history. All participating individuals provided written consent prior to inclusion.

### 2.2 Surgical cohort for the GWAS discovery phase

For the initial discovery GWAS, aimed at identifying SNPs associated with VTE events up to 30 days after surgery, a cohort consisting of all patients with at least one surgical procedure in the UK Biobank was identified. Surgical history was identified using OPCS4-codes from UK Biobank data field 41200. All procedures with at least one VTE-event were included. Patients with a 30-day postoperative VTE event were used as cases and those without as controls. VTE events were identified using *International Statistical Classification of Diseases, 10*^*th*^ *revision* (ICD-10) and ICD-9 codes. The identifying ICD-10 codes for VTE were I260, I269, I801, I802, I803, I808, I809, I820, I821, I822, I823, I828, I829, O082, O223, O871, O882 and I81 and The identifying ICD-9 for VTE were 4151, 4511, 4512, 4519, 4531, 4532, 4534, 4538, 4539, 4534, 4531, 4532 and 4539 similar to the work by Sultan et al. [17]. Phenotypic data on obesity and hypertension was identified by ICD-10 codes. Information on malignancy was identified using the UK biobank data fields 2443 and 2453, respectively. Information on tobacco use was defined as current smoker at inclusion or previous smoker using data fields 1239 and 1249, respectively. Information on diabetes was extracted from data fields 2443.

### 2.3 Non-surgical cohort for the replication GWAS

For the second analysis step, we identified all patients in the UK Biobank with available genomic information excluding patients in the discovery surgical cohort. As such, we used two unique cohorts with no overlap between participants. In this cohort, all patients with a registered VTE event as defined above at any time were used as cases, with the remainder serving as controls.

### 2.4 Genotyping data and quality control

The first 50,000 study participants were genotyped using the Affymetrix UK BiLEVE Axiom array while the remaining 450,000 study participants were genotyped using the Affymetrix UK Biobank Axiom array which genotyped approximately 850,000 Single Nucleotide Polymorphism (SNP) variants. No major differences exist between the two types of arrays.

Only genotyped non-imputed variants were considered in this analysis. Quality control was performed with standard approaches using PLINK v1.90b6.16 (Shaun Purcell, MA, US) [18,19]. Patients with sex discrepancies, cryptic relatedness, outlying heterozygosity rates and patients with a missing genotype rate of 2% were excluded. Furthermore, markers with missingness rate of 2% and markers very unlikely to be in Hardy-Weinberg equilibrium were excluded. Only variants with a minor allele frequency (MAF) of more than 5% were considered in the final analysis.

### 2.5 Statistical analysis

The GWAS-analyses were conducted with a mixed linear model (MLM)-based approach as fastGWA with a sparse genetic relationship matrix (GCTA version 1.93.2 beta for Windows) [20,21]. A p-value of 5 × 10^−8^ was considered statistically significant. The Manhattan and QQ-plots were generated using R package qqman (R version 4.0.2) [22]. SNPs are denoted using the Reference SNP cluster ID (rs) [23,24].

For both the discovery and replication GWAS, we performed two separate analyses. The first was a univariate model, associating SNPs with VTE events. The second was an adjusted model, controlling for relevant confounders including sex, age, smoking, obesity open/minimal invasive surgery (surgical discovery GWAS only), diabetes, history of cancer and hypertension.

Information on thromboprophylaxis regimens used in the discovery (surgical) cohort was not available in the UK Biobank. Demographic data is presented as means with standard deviations (SD) or percentages where appropriate.

## 3 Results

### 3.1 Discovery surgical GWAS

Prior to quality control, a total of 502,505 patients with available genomic data were identified. After quality control and restricting the analysis to patients with a history of the selected surgical procedures, 140,831 patients with 254,068 SNP variants were left for the discovery GWAS analysis. Baseline characteristics are listed in Table 1. Overall, 799 patients had a recorded VTE event within 30-days of a procedure, yielding an incidence of 0.57%.

**Table 1:** Baseline characteristics for discovery surgical GWAS (top) and general replication cohorts (bottom). Data presented as means with Standard deviations (SD) or percentages where appropriate.

Surgical discovery cohort	Cases	Controls
Number, N	799	140,032
Age ± SD, years	59.9 ± 7.0	58.1 ± 7.8
Female, n (%)	393 (49.2)	78,558 (56.1)
Obesity, n (%)	83 (10.3)	8925 (6.3)
Current or past smoker, n (%)	329 (41.2)	56,523 (40.4)
Major surgery, n (%)	667 (83.5)	109,656 (78.3)
**General replication cohort**	**Cases**	**Controls**
Number, N	6260	355,414
Age ± SD, years	59.3 ± 7.3	55.9 ± 8.1
Female, n (%)	2812 (44.9)	163,794 (46.0)
Obesity, n (%)	581 (9.2)	9636 (2.7)
Current or past smoker, n (%)	2521 (40.2)	128,398 (36.1)

**Fig. 1:**
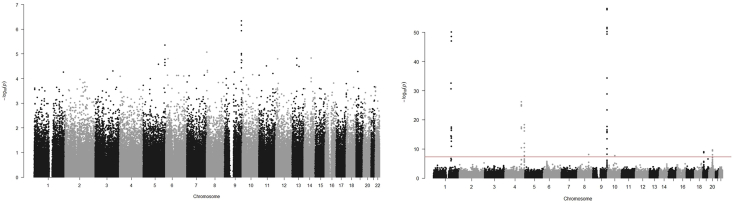
Manhattan plot for discovery genome wide association study for venous thromboembolism 30-days post-surgery (surgical discovery cohort, Figure A) and in the general population (replication cohort, Figure B).

**Fig. 2:**
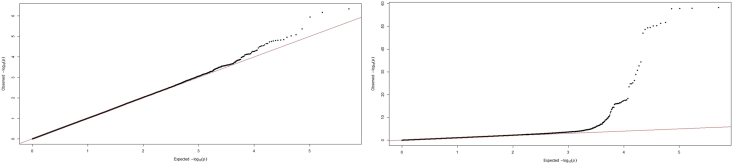
Quantile-Quantile plot for discovery genome wide association for venous thromboembolism 30-days post-surgery (surgical discovery cohort, Figure A) and in the general population (replication cohort, Figure B).

The top 20 SNPs are shown in Table 2. No variants met the predefined threshold of significance (p < 5 × 10^−8^). The rs505922 SNP was the top candidate (p = 4.55 × 10^−7^). The variant is an intronic variant in the *ABO gene*. The variant was in linkage disequilibrium (LD) with several variants in the same locus below p = 5 × 10^−7^, indicating a non-random association of alleles and thus a shared pattern of inheritance. The locus has previously been associated with VTE, both in the UK Biobank and in independent cohorts [13,14,25]. The genomic inflation factor lambda was calculated at 1.02. The Manhattan plot depicted in Fig. 1A and the QQ-plot for expected and observed p-values is depicted in Fig. 2A. The adjusted model showed similar results, with no SNPs reaching genome wide significance, but with the same top SNPs identified.

**Table 2:** Top 20 Single Nucleotide Polymorphisms with crude and adjusted P-values from the surgical discovery cohort.

SNP	Crude P-value	Adjusted P-value	Nearest gene	Exon/Intron/Intergenic
rs505922	4.55 × 10^−7^	3.33 × 10^−7^	*ABO*	Intron
rs612169	6.71 × 10^−7^	4.77 × 10^−7^	*ABO*	Intron
rs687621	1.15 × 10^−7^	8.34 × 10^−7^	*ABO*	Intron
rs72815442	4.35 × 10^−6^	4.07 × 10^−6^	*DRD1*	Intergenic
rs13280606	8.41 × 10^−6^	7.93 × 10^−6^	*CSMD1*	Intron
rs8176719	9.58 × 10^−6^	7.00 × 10^.6^	*ABO*	Intron
rs643434	1.11 × 10^−5^	7.79 × 10^−6^	*ABO*	Intron
rs72733608	1.45 × 10^−5^	1.48 × 10^−5^	*ESRRB*	Intergenic
rs9597136	1.50 × 10^−5^	1.54 × 10^−5^	*PCDH17*	Intergenic
rs6899869	1.54 × 10^−5^	1.29 × 10^−5^	*ID4*	Intergenic
rs34362840	1.58 × 10^−5^	1.61 × 10^−5^	*PSMC1P8*	Intergenic
rs11749035	1.68 × 10^−5^	1.59 × 10^−5^	*DRD1*	Intergenic
rs657152	1.76 × 10^−5^	1.26 × 10^−5^	*ABO*	Intron
rs1112786	1.85 × 10^−5^	1.98 × 10^−5^	*ADARB2*	Intron
rs507666	2.23 × 10^−5^	1.82 × 10^−5^	*ABO*	Intron
rs2168631	2.24 × 10^−5^	2.24 × 10^−5^	*DRD1*	Intergenic
rs11956605	2.60 × 10^−5^	3.20 × 10^−5^	*ZNF474*	Intron
rs73191601	2.82 × 10^−5^	3.13 × 10^−5^	*PCDH17*	Intergenic
rs11746641	2.91 × 10^−5^	2.78 × 10^−5^	*DRD1*	Intergenic
rs61896704	3.03 × 10^−5^	3.13 × 10^−5^	*INCENP*	Intergenic

### 3.2 Replication GWAS in cohort of surgery naïve patients

After quality control and restricting the analysis to patients with no history of the selected surgical procedures, 305,349 patients with 254,068 SNP variants were left for the replication GWAS analysis. Baseline characteristics are listed in Table 1 and the top 20 SNPs listed in Table 3. The trending variant in the *ABO* gene from the discovery GWAS, rs505922, was also found to exhibit the strongest association with VTE (p = 6.0885 × 10^−59^) in the general population. Several variants in the same locus were also significantly associated with VTE. Variants in other loci were also found to be associated with VTE, albeit these did not align with the top associations in the surgical cohort. The genomic inflation factor was calculated at 1.06. The Manhattan plot is depicted in Fig. 1B and the QQ-plot for expected and observed p-values is depicted in Fig. 2B. Adjusting for confounders did not alter results.

**Table 3:** Top 20 Single Nucleotide Polymorphisms with crude and adjusted P-values from the general replication cohort.

SNP	Crude P-value	Adjusted P-value	Gene	Exon/Intron/intergenic
rs505922	6.09 × 10^.59^	2.43 × 10^−59^	*ABO*	Intron
rs612169	1.25 × 10^−58^	4.49 × 10^−59^	*ABO*	Intron
rs687621	1.62 × 10^−58^	9.13 × 10^−59^	*ABO*	Intron
rs507666	1.82 × 10^−58^	3.64 × 10^−58^	*ABO*	Intron
rs8176719	2.10 × 10^−52^	2.58 × 10^−52^	*ABO*	Intron
rs643434	5.13 × 10^−52^	1.96 × 10^−52^	*ABO*	Intron
rs657152	5.83 × 10^−51^	2.53 × 10^−51^	*ABO*	Intron
rs6687813	6.62 × 10^−51^	2.50 × 10 ^−51^	*F5*	Intergenic
rs651007	3.95 × 10^−50^	9.54 × 10^−50^	*ABO*	Intergenic
rs579459	4.09 × 10^−50^	1.09 × 10^−49^	*ABO*	Intergenic
rs2420371	2.44 × 10^−49^	1.12 × 10^−49^	*F5*	Intron
rs1018827	8.92 × 10^−48^	2.41 × 10^−48^	*F5*	Intron
rs56342119	4.14 × 10^−35^	4.54 × 10^−35^	*SURF4*	Intron
rs75112989	2.57 × 10^−33^	1.92 × 10^−33^	*ATP1B1*	Intron
rs1208134	2.18 × 10^−31^	9.09 × 10^−32^	*CCDC181*	Intron
rs581107	1.45 × 10^−29^	1.97 × 10^−29^	*ABO*	Intron
rs2066865	6.81 × 10^−27^	2.93 × 10^−27^	*FGG*	Intergenic
rs6050	7.15 × 10^−26^	3.56 × 10^−26^	*FGA*	Exon
rs7681423	1.54 × 10^−25^	5.33 × 10^−26^	*FGG*	Intergenic
rs13109457	1.80 × 10^−25^	8.75 × 10^−26^	*FGA*	Intergenic

## 4 Discussion

In this analysis, we report the findings of potential genetic risk factors for postoperative VTE events. No variants in the surgical discovery cohort reached genome-wide significance. However, the top variants from the discovery cohort were replicated with genome-wide significance in a unique cohort of VTE in the general population. The top candidate in both cohorts was rs505922 (position GRCh38. p12: 133,273,813), with several other candidate SNPs in or near the *ABO* gene on chromosome 9 reaching genome-wide significance in the replication cohort. These results indicate that these SNPs may modulate VTE risk, although genome-wide significance could not be demonstrated in the surgical setting. Interestingly, the fact that association rankings of other SNPs did not completely overlap between the surgical and non-surgical cohorts could suggest that the influence of genomic variants in the surgical setting cannot be directly extrapolated from findings in non-surgical cohort. Adjusting for relevant confounders did not alter these findings.

The *ABO* gene on chromosome 9 encodes a glycosyltransferase protein which determines the human ABO blood type [26,27]. Variants in the gene are associated with a wide range of pathologies ranging from myocardial infarction to Grave's disease and have recently been associated with the severity of SARS-CoV-2 infections [[28], [29], [30]]. Furthermore, they have previously been associated with VTE in different patient populations and are widely accepted as a risk factors [[31], [32], [33]].

The intronic variant rs505922_C is highly prevalent with an overall frequency of 35% with similar rates worldwide [34]. This specific *ABO* gene SNP has previously been associated with myocardial infarction [35]. Results from a GWAS on pancreatic cancer risk showed that the more common T allele, which is in LD with a base pair deletion coding for the O-antigen in *ABO*, had a protective association with pancreatic cancer [36]. In our cohort the more common T allele of rs505922 was also protective against postoperative VTE compared to the C allele.

A variant in LD with the top variant was rs8176719_TC, which was significantly associated with VTE in the replication cohort. The variant is an insertion/deletion-variant with the deletion as the most common (frequency = 64% worldwide) and the insertion of C as the least common variant (frequency = 36% worldwide). European frequencies are the same [34]. The variant is one of the major base pairs determining a patients ABO blood type [27]. The insertion of the C is highly correlated to the A or B blood type. If a patient is homozygote for the deletion, they are most likely to be blood type O. If a patient is heterozygote for the insertion, they are most likely blood type A or B, and if they are homozygous for the insertion, they are most likely to be A, B or AB. The SNP does not determine the Rh antigens [27]. As all the associated variants in *ABO* in our cohort lie close, they are probably in LD. It is likely that only one SNP is the causative agent in the association with postoperative VTE. D’ values, ranging from 0 to 1 with higher values indicating a stronger linkage and thus coinheritance of alleles from LDlink, are shown in Supplementary Table 1. Using available reference haplotypes of the 1000 genomes project point to a high coinheritance between some of associated SNPs, suggesting that all of them are in LD [37,38].

Due to the LD observed, there is thus likely a co-inheritance between the top SNP and other *ABO* gene SNPs, indicating that the causative SNP for VTE risk could rs8176719, pointing to a possible association between ABO-type and postoperative VTE. The risk allele is C, which is highly correlated with blood type A and B meaning that patients with these blood types may be at a higher risk of postoperative VTE compared with patients who are homozygote for the deletion and thus most likely blood type O. Indeed, ABO blood group has previously been strongly associated with VTE with studies pointing to a risk increase of up to 60% being non-O compared with blood group O [39,40]. However, more recent research suggests that the risk stratification between blood groups is more complex, and that antigenic subgroups between A, B and O alleles may differentiate the risk [40,41].

The pathophysiology behind the association between the non-O blood group and postoperative VTE may be mediated through von Willebrand factor (vWF) which appears to be a mechanism in other patient populations [40,42]. It is believed that the presence of A, B and H(O) antigens on vWF have an impact on the clearance of vWF, although the mechanism is poorly understood [43,44]. Lower clearance of vWF in non-O individuals and the association between higher vWF-levels and thromboses may explain the association between the TC allele of rs8176719 and postoperative VTE. Thus, the association between non-O blood groups and postoperative VTE may be explained by similar mechanisms as previously described. However, other mechanisms more specific to the postoperative stress response may exist, and future research needs to determine the role and pathophysiology of common variants in the *ABO* gene and blood groups on postoperative VTE risk.

Collectively, these findings thus provide insights into genomic mechanisms underlying the previously observed associations between ABO blood group and VTE events [39,40], and furthermore suggests that while considerable overlaps exist, SNPs identified in non-surgical cohorts can only partly be replicated in the surgical setting.

Genotyping for VTE prophylaxis is not part of the standard preoperative assessment in most health care systems, except for specific indications (e.g., history of multiple VTE events), but the presented results indicate a potential for incorporating this modality, at least for high-risk surgical procedures. However, it is important to emphasize that the results of this study do not currently present an indication for preoperational screening of the presented variants, but they do suggest that replicating this analysis in larger genotyped surgical cohorts could be indicated.

One may wonder why common genetic risk factors for VTE, like the factor V Leiden variant, rs6025, were not significant in our analysis. While the possibilities are many, a very plausible explanation is that these mutations were already diagnosed preoperatively, possibly from previous VTE events, and that these patients were treated on a more aggressive antithrombotic regimen postoperatively.

### 4.1 Limitations

Our study has limitations. First, the study may be underpowered to detect associations between SNPs and VTE events. Indeed, with the observed VTE incidence of 0.57% in the surgical cohort, an assumed genotype relative risk of 1.5 and a disease allele frequency of 0.3, a post-hoc power calculation indicated that 1250 postoperative VTE patients would be required to identify an association at the genome wide significance level with a power of 80%. As such, the 799 patients identified in this study represents insufficient study power and the results should be interpreted with this in mind. Furthermore, the choice of proceeding with the validation step in the general cohort rests upon this fact, as it provides further information to support the relevance of the identified top-candidate genes in the surgical cohort, even if lacking genome-wide significance.

Secondly, this is a retrospective case-control study, and hence no causation can be drawn. Thirdly, confounders not accounted for in the model could have affected results. Notably, the available data did not allow for the incorporation of the thrombophylactic regimen used in the perioperative phase into the predictive models, which is likely to impact the VTE risk. We did, however, chose surgical procedures performed in the context of a modern health care system, where updated prophylaxis guidelines are likely to have been adhered to for the majority of patients. Further, the results should be interpreted in the context of the current literature on the relationship between *ABO* and VTE. This gives reassurance that the bias from the lack of data on thromboembolic medications may be limited. Lastly, to our knowledge, this is an inherent limitation in most major biobanks and genetic studies on surgical thromboembolisms will therefore be difficult to conduct without this lack with the data available in the world at this moment*.* Nevertheless, this is a major limitation of the data, and future studies on the genetic risk of postoperative VTE with the inclusion of thrombophylactic medication data, when available, are warranted.

The discovery and replication cohorts are unique but are from the same dataset, which may compromise an inherent bias. Ideally, findings from this study should be externally replicated in an independent cohort.

Another limitation of our study is the definition of VTE in which we use ICD-10 codes, which likely represents a significant underreporting. This definition will exclude most asymptomatic VTEs, which may have put cases in the control category. Also, as is the case for any retrospective study, coding errors could have affected results.

## 5 Conclusion

In conclusion we report that no variants reached genome-wide significance for VTE 30 days after surgery in a cohort of surgical patients. However, the top candidate (rs505922) and other SNPs in the *ABO* gene was found to be highly associated with VTE in a unique cohort of the general population, providing a potential genomic mechanism for the previously observed association between blood type and VTE risk. Furthermore, results indicate that SNP and VTE associations may not be identical between surgical and non-surgical patients.

## Funding

Funded by a grant from the 10.13039/501100009708Novo Nordisk Foundation to MS (Grant # NNF19OC0055183).

## Provenance and peer review

Not commissioned, externally peer-reviewed.

## Research registration unique identifying number (UIN)

Name of the registry: ClinicalTrials.gov

Unique Identifying number or registration ID: #NCT04940377

Hyperlink to your specific registration (must be publicly accessible and will be checked): https://clinicaltrials.gov/ct2/show/NCT04940377

## Ethical statement

Access to the UK biobank data was approved by the UK biobank consortium (Study ID #60861). Under Danish law, the study was exempt from local ethics approval due to the de-identified nature of the dataset.

## Author contribution

**MC**: Conceptualization; Data curation; Formal analysis; Investigation; Methodology; Resources; Software; Visualization; Roles/Writing - original draft.

**AB**: Conceptualization; Data curation; Investigation; Methodology; Software; Supervision; Visualization; Roles/Writing - review & editing.

**MS**: Conceptualization; Data curation; Funding acquisition; Investigation; Methodology; Project administration; Resources; Software; Supervision; Validation; Roles/Writing - review & editing.

## Guarantor

Martin Sillesen serves as the Guarantor of the study.

## Declaration of competing interest

The authors declare no conflicts of interests.
