# Supplementary material for: An assessment of the effect of the genotype on postoperative venous thromboembolism risk in 140,831 surgical patients
Source: Ann Med Surg (Lond). 2021 Oct 13;71:102938. doi: 10.1016/j.amsu.2021.102938 (PMC8577341; doi:10.1016/j.amsu.2021.102938)
Supplement: Multimedia component 1 [file mmc1.docx]

**SUPPLEMENTARY DATA**

**Supplementary Table 1**: D’ values from LDlink using publicly available haplotypes from 1000 genomes project. All SNPs in the table are paired with rs8176719.

D’value: D' values range from 0 to 1 with higher values indicating tight linkage of alleles

| **SNP** | **D’-value** |
| --- | --- |
| rs687621 | 0.94 |
| rs612169 | 0.9064 |
| rs505922 | 0.8957 |
| rs643434 | 0.9557 |
| rs657152 | 0.9562 |

| **SNP** | **D’-value** |
| --- | --- |
| rs687621 | 0.94 |
| rs612169 | 0.9064 |
| rs505922 | 0.8957 |
| rs643434 | 0.9557 |
| rs657152 | 0.9562 |

| **SNP** | **D’-value** |
| --- | --- |
| rs687621 | 0.94 |
| rs612169 | 0.9064 |
| rs505922 | 0.8957 |
| rs643434 | 0.9557 |
| rs657152 | 0.9562 |
